# Facilitators and barriers in prevention of cardiovascular disease in Limpopo, South Africa: a qualitative study conducted with primary health care managers

**DOI:** 10.1186/s12872-021-02290-1

**Published:** 2021-10-12

**Authors:** Mbuyiselo Douglas, Nancy Kgatla, Tholene Sodi, Geofrey Musinguzi, Tebogo Mothiba, Linda Skaal, Mpsanyana Makgahlela, Hilde Bastiaens

**Affiliations:** 1 Faculty of Humanities and Health Sciences, University of Limpopo, Sovenga, South Africa; 2 Primary and Interdisciplinary Care, University of Antwerp, Antwerp, Belgium

**Keywords:** Cardiovascular disease, Facilitators, Barriers, Prevention, Primary health care managers

## Abstract

**Background:**

In the Southern African countries, cardiovascular disease burden is increasing and the second most prevalent cause of death after infectious diseases. The sustainable primary prevention of cardiovascular disease is associated with the engagement of facilitators that support it and hindered by barriers that undermine the support of a healthy lifestyle at the community level. The purpose of the study was to investigate facilitators and barriers at the level of primary health care facilities, on prevention of cardiovascular disease in Limpopo Province of South Africa.

**Methods:**

This study is an exploratory and descriptive qualitative design, where open-ended key informant interviews were conducted among 20 primary health care managers conveniently sampled in their respective health care facilities. Coding and analysis were done using the thematic analysis method with the assistance of Atlas ti qualitative software.

**Results:**

Various facilitators for the prevention of CVD were identified in this study. One of such facilitators is the availability and adherence to CVD treatment guidelines in the district. Other facilitators included top-down health education programme; collaboration with schools, traditional and religious leaders; the use of modern technology; and a structured healthcare system. Barriers were also identified as poor infrastructural development; shortage of medical supplies and equipment; lack of health promotion activities; shortage of nurses and other health care personnel; and poor accessibility to primary health care services.

**Conclusion:**

This study has identified barriers and facilitators that may be harnessed to improve cardiovascular disease prevention, care, and management in a rural setting in South Africa. The facilitators should be strengthened, and barriers identified redressed.

*Trial registration number*: REC-0310111-031.

**Supplementary Information:**

The online version contains supplementary material available at 10.1186/s12872-021-02290-1.

## Background

Currently, the burden of cardiovascular disease (CVD) in Africa is growing and changing in nature [1, 2]. From the predominance of rheumatic heart disease and cardiomyopathies 50 years ago to hypertensive heart disease and haemorrhagic stroke increasing progressively in the past 25 years [1]. Among non-communicable diseases (NCDs), the four top killers that together account for more than 80% of all premature NCDs deaths include CVD, cancers, respiratory diseases, and diabetes [3]. Studies estimates that the burden of CVD has doubled from 1990 to 2020 (1,2) CVD account for 82% of global mortality and 88% of mortality and morbidity from CVD low- and middle-income countries [2, 4]. A study conducted by Nojila et al. [2] stated that Black South Africans are experiencing relatively high age-standardised death rates (ASDRs) from cerebrovascular disease, hypertensive heart disease, diabetes mellitus, ischaemic heart disease (IHD) and cardiomyopathy compared to Coloureds, Asians, and Whites.

The dynamics are changing, and the World Health Organization (WHO) has projected that by the year 2030, CVD will surpass communicable diseases as the leading cause of death in the African continent [4]. People in low and middle-income settings often do not have the benefit of integrated primary health care (PHC) programmes for prevention, early detection and treatment of CVD compared to those in high-income settings [4]. As a result, some people in low and middle-income countries are often detected late in the course of the disease and they die younger from CVD and other NCDs in their most productive years. For instance, in 2013, an estimated one million premature deaths were attributed to CVD in Sub-Saharan Africa, constituting 5.5% of all global CVD-related deaths and 11.3% of all deaths from different causes [5].

Furthermore, out of the 17 million global NCDs related deaths that occurred in 2015, 82% were in low and middle-income countries, including Sub-Saharan Africa, out of which 37% were caused by CVD [4]. Therefore, because of the increase in the number of CVD cases, stroke and heart failure have become common in low to middle-income in the Sub-Saharan Africa region [6]. South Africa is currently suffering from the burden of infectious diseases and NCDs that creates a significant impact in the South African health care system [2, 7]. NCDs inclusive of CVD accounted for 57.4% of deaths while communicable diseases were responsible for 31.3% of deaths in 2016 [4].

A systematic review of (eligible) 28 studies identified several facilitators and barriers related to prevention of CVD including structural, organisational, professional, patient-related factors and attitudinal components [8]. Facilitators that support or barriers that hinder the implementation of interventions supporting health lifestyle in PHC facilities, have been identified [9]. Therefore, before implementing a CVD preventive programmes in PHC, it is important to identify potential facilitators and barriers that may exist among primary care health care facilities and the population [8–10].

The purpose of this study was to investigate facilitators and barriers at the level of primary healthcare facilities, in prevention of cardiovascular disease in Limpopo Province of South Africa.

## Methods

### Study design and participants

The study utilised an exploratory and descriptive qualitative design, key informant (KI) interviews were used to investigate the facilitators and barriers of CVD prevention. The study participants were 20 PHC managers selected using convenience sample from the 11 primary health care facilities. The study comprised fifteen (15) participants from one rural setting and five (5) one semi-urban area. The key informants were qualified clinical nurse practitioners with vast experiences in PHC management positions. The participants encompassed two levels of management ranks, 4 local area managers, and 16 operational managers, overseeing and managing the 11 PHC facilities respectively (Table 1).

**Table 1:** Demographic characteristics of participants

Primary health care managers	Participants (n = 20)			
	Local area managers (n = 4)	%	Operational managers (n = 16)	%
*Gender*				
Male	2	50	1	6.25
Female	2	50	15	93.75
*Mean age (years)*				
Male	52	–	48	–
Female	55	–	47	–
*Qualifications*				
Diploma	2	50	10	62.5
Degree	2	50	6	37.5
*Ethnicity*				
Sepedi	3	75	12	75
Others (Xitsonga or Venda)	1	25	4	25

### Setting and population

The study was conducted at Polokwane Municipality in Capricorn District of Limpopo Province. The population of Polokwane municipality is 797 127 [2016] with 80.36% speaking Sepedi local language [11]. Polokwane (previously called Pietersburg) is the capital town of Limpopo which is one of the nine provinces of South Africa. Limpopo ranks fifth position in South Africa in both surface area and population, covering an area of 125 754km^2^ and being home to a population of 5 779 090. The district was chosen for the study due to the myriad of health challenges that it faces, including the burden of CVD [12].

### Data collection

An open-ended key informant interview guide was used to collect the data from the participants (Additional file 1: Table S1). The guide solicited participants’ views and experiences with the management and prevention of CVD in their various PHC facilities and local areas. The questions focused on the current situation and elaborated on facilitators and barriers that exist and could be utilised to improve the prevention and management of CVD. The KI guide also contained questions on the demographic characteristics of participants, prevention, care, and management. The interviews were conducted in English. After each interview, the lead investigator (MD) and an assistant researcher (NK) made notes and discussed the preliminary observations. This initial analysis helped to improve the wording of the questions in subsequent interviews as well as in noting of areas that needed to be emphasised. The interviews took place from March 2018 to April 2018 at the work premises of the participants. Participants were given unique codes; OPM (#) for operational managers and AM (#) for area managers, to conceal their identities and to be able to trace the source of each transcript for the purpose of member checking. However, only the lead investigator knew the exact participants to which the codes/transcripts belonged to ensure anonymity and confidentiality. The interviews were audio-recorded utilising a digital voice recorder.

### Data analysis

All interviews were transcribed verbatim. Coding and analysis were then done using the thematic analysis approach with the assistance of the Atlas.ti qualitative software. The inductive coding process was followed in coding the data. Thus, codes were derived from original narratives and ideas expressed by the participants [13]. This was so because we were interested in identifying past experiences of the PHC managers in relation to the facilitators they faced in the prevention of CVD as well as the barriers that existed and could be explored in the prevention and management of the condition. The coding frame was developed through an iterative process of multiple discussions between the authors by paying special attention to similarities and differences. The next stage of analysis involved linkages between codes, themes, appropriate quotes, and the study objectives. Two considerations informed the interpretive analysis of codes and themes. First, the facilitators that participants faced in the prevention and management of CVD and second, the barriers existed for the prevention and management of CVD in the Capricorn District. The participants identified five facilitators and five barriers (Table 2). Appropriate excerpts for each theme were used to support our findings. Inductive thematic saturation was deemed to have been achieved when there were non-emergence of new codes or themes [14]. Data saturation was reached at participant number 19, but interviews were stopped at participant number 20 because the last participant was waiting patiently for her turn to be interviewed. Member checking was conducted by returning the results to participants to check for accuracy and resonance with their experiences.

**Table 2:** Categories, themes, and quotes

Categories	Themes	Quotes
Facilitators	Policies and guidelines for the prevention of CVD	*We do have policies, although they are produced as guidelines. The guidelines are there to lead us on how to manage and prevent the diseases we are talking about. Type 1 diabetes, types 2 diabetes and hypertension have their own national guidelines separately (Participant # 15, OPM)* *We do have set of operating procedures that guide us. We also have the protocol of essential drug list that guides us on how to go about doing the assessment of chronic diseases and initiating treatment for the new cases (Participant # 12, OPM)*
	Health education programme	*Health education is the basic in every area at the primary health care level, so we give health education to community members concerning healthy lifestyle for them to be able to avoid these kinds of diseases called CVD (Participant # 1, OPM)* *Every morning what we provide health education on prevention of some diseases including CVD before we start daily routine. Still, I think it is not enough, it would be good if we can start going to the communities regularly* (*Participant # 5, OPM*)
	Collaboration with other stakeholders	*We have an exceptionally good relationship with the traditional leaders around here where our health facilities are. Usually, whenever we have problems, we first go to the traditional leaders; the traditional leaders have regular meetings, some on Tuesdays, and others on Thursdays. That is where we discuss health issues, and they share with us theirs concerns and together we find a way forward on how to deal with the problems related to CVD, so I think their involvement is a good strategy* (Participant # 1, AM) *The traditional leaders are usually invited to address the people gathered in the funerals because many community members attend funerals in masses. We are usually given a slot with the traditional leaders to address the community about prevention of CVD* (Participant # 3, AM) *There is a clinic that belongs to ZCC church. The church is the one that owns the building; they have donated it to the Department of Health. They are the ones who are responsible in assisting us with maintenance and other necessary developments. They are particularly important in the provision of healthcare to our people, and I think we need the involvement of more churches like this to work together in fight against CVD (Participant # 14, OPM)* *The churches are contributing a lot, where there are no halls, we use churches for Centralised Chronic Medicines Dispensing and Distribution (CCMDD). In the past, it was a challenge when other churches did not believe in medication, but now there is a change, the pastors give us slot during their church services to address the people on prevention of CVD. Some churches also call health professionals to come and provide health education sessions during their seminars and conferences (*Participant # 2, AM*)* *In this area we are working with the NGOs and they are contributing a lot. We utilise them for home-based care to manage CVD and other conditions. They usually go to the communities for home visits, even when nurses go out for door-to-door campaign for health awareness and sometimes we work with them* (Participant # 8, OPM) *The NGOs are playing an especially important role in the prevention of CVD. For example, we do have NGOs that help with home-based care. The youth groups, the clinic committee and ‘indunas’ (community volunteer groups) are so involved in the health-related issues* (Participant # 7, OPM)
	The use of modern technology	*We have got computers and mobile phones. The clients receive mobile phone messages and calls to collect their medication from the health care facilities. The mobile phones are also instrumental to trace clients who are defaulting treatment. Health care workers utilise mobile phone to communicate with most patients on chronic treatment even by visiting them in their farms* (Participant # 2, OPM) *We have computers, although we are still learning to use them, some staff members can do so. This has made data capturing easy and help us to know the actual number of clients (head count) on treatment and those who will be starting their treatment soon* (Participant # 13, OPM)
	A structured healthcare system	*There is a director at the provincial level who is leading the management of chronic conditions including CVD, at the district level we have a deputy director. There is an acting deputy director at a district level who is leading the management of chronic diseases around 21 clinics in Polokwane Municipality. In my office as a sub-district manager, I report to the deputy director, we have operational managers at a facility level and clinical nurses below the operational managers* (Participant # 3, AM) *We have professional nurses: A male nurse, who is the coordinator of chronic diseases, and a lady professional nurse who coordinates diabetes and hypertension. Another nurse coordinates HIV/AIDS and sexually transmitted infections (HAST). Sub-districts report to the district and district to the province. The province reports to the National Department of Health (*Participant # 1, AM*)* *We open at 07h00 and close at 18h00 for access of all people even those who go to work can be afforded primary health care services* (Participant # 9, OPM) *We operate from 07h00 to 18h00, but we can see all the patients that come in according to ideal clinical practice, anybody who comes here in the facility must be seen before we close the facility* (Participant # 16, OPM) *We are having a very dedicated committed team in the nursing section that manages chronic diseases in our facility. No matter how many the patients are, the team attends to them all* (Participant # 2, OPM) *We also have enrolled nurses (staff nurses) that are assisting clinical nurse practitioners. Despite their workload, they see to it that our clients are properly cared for. Without them, the fight against CVD would have been lost long ago* (Participant # 4, OPM)
Barriers	Poor infrastructural development	*The structure is too small we do not even have privacy for patients when we take vital signs and interviews because we do not have a room for these kinds of procedures. Where we put our files is also where we take vital signs* (Participant # 5, OPM) *Yes, infrastructure is a barrier as you can see that our infrastructure is too small during days for consultations for chronic conditions, you find that the place is just packed all over, overcrowded with few chairs for patients to sit outside”* (Participant # 2, OPM) *No, the infrastructure does not facilitate the prevention of CVD, it is an exceedingly small clinic and the clients sometimes undermine our capacity due to its size (*Participant # 11, OPM)
	Shortage of medical supplies and equipment	*We do sometimes experience shortage of treatment [medication]. Sometimes when patients come, we give them incomplete prescriptions because of shortage. We usually do this when we are running short of medication* (Participant # 6, OPM) *In this facility, we do not have enough BP machines and glucometers. Yes, we may have some glucometers, but you will find out that there are only 2 glucometers for the entire facility to share in 4 consulting rooms, so sometimes we rely on history taking (*Participant # 1, AM) *The electronic BP machines we have are designed by the manufacture to check few numbers of patients at a time, but now imagine a nurse must take BP for 20 patients, which end up giving the wrong readings. So, some patients end up being diagnosed falsely* (Participant # 16, OPM)
	Lack of health promotion activities	*We are having a health promotion manager who coordinates health promotion programmes. She is located at the district office. The challenge is that she only visits the communities on invitation during special calendar events. No health promotion practitioner is available at the sub-district and local levels. We encourage our clinics to liaise with her to organise imbizo (community meetings) and awareness campaigns where the manager for health promotion comes in. She usually assists in terms of preparation for those awareness campaigns by supplying posters, reading material and handouts. But the health behavioural change activities that are essential are not dealt with daily* (Participant # 4, AM) *Unfortunately, the health care system now is not the same as it was in the 1980s and early 1990s. This is because in the previous decades mentioned, you would see community health nurses, specifically going out to do community outreach services. This is political, hee hee (laughing). Let us say, in the mobile teams we used to have a team specifically for schools, the mobile would have a specific team for TB. They were having their own vehicles and they specialised in those services. Those teams were disbanded due to poor management. I think they have seen that the old system was functional. That is why they are now trying to bring those teams back* (Participant # 3, AM) *Health promotion activities are usually limited with no capacity to deal with the predisposing factors of CVD such as obesity and lack of physical activity* (Participant # 2, AM)
	Shortage of nurses and other personnel	*Serious challenges are at the clinic facility level. Nurses are understaffed. People retire, people die, and they are not replaced. We identify vacant post for advertisements, we write to the Human Resource section to motivate for additional posts, but there was a red tape since 2012 up to now. No retired nurse has been replaced. This is a political decision that hinders prevention, care, and management at the clinics. In my area of work, I have 3 acting operational managers. At least 4 OPMs have been appointed long ago and they are due to retire soon. Two of them will be retiring this year* (Participant # 4, AM) *Unfortunately, there is no visiting medical doctor in our clinic. We refer our patients who need to see the doctor to Mankweng Hospital. The pharmacy assistant visits every Wednesday, and the physiotherapist, speech, and hearing therapist maybe once a month. We do not have an environmental officer and a health promotion practitioner. We liaise with the district if we seriously need them. The dietician that we had unfortunately resigned. So, for now we do not have, but other facilities do have dieticians and visiting medical doctors* (Participant # 15, OPM) *We do not have an administrative officer to capture those files there. We do not have enough staff that is why you see today’s situation. I am the only nurse who was on duty* (Participant # 15, OPM) *The first one is shortage of staff, I am working here at this clinic, at the same time I should manage 5 catchment areas, how is that possible?* (Participant # 2, OPM)
	Poor accessibility to PHC services	*The opening hours are not conducive and primary health care is not accessible because we are only opening for 10 h in a day from 7 am to 6 pm. During the night, the facility is closed, so if a patient has a problem at night, the family will have to carry him/her to the hospital (Participant # 11, OPM)* *The family calls the ambulance to where they stay, or they hire a neighbour’s car to carry their member the hospital during the night. Our facility is not opened to operate at night, so I trying to say that accessibility to healthcare services is a problem in this area (Participant # 8, OPM)*

## Results

### Facilitators and barriers

Five facilitators in CVD prevention and management were identified as themes. These include: the availability of policies and guidelines for the prevention and management of cardiovascular; health education programme; collaboration with schools, traditional and religious leaders; the use of modern technology and a structured healthcare system. Five key barriers were also identified as themes that affect the prevention and management of CVD in the district. These are: poor infrastructural development; shortage of medical supplies and equipment; lack of health promotion activities; shortage of nurses and other health care personnel; and poor accessibility to primary health care services.

## Discussion

### Facilitators of CVD prevention and management

Various prevention and management facilitators for CVD were identified in this study. One of such facilitators is the availability and adherence to CVD treatment guidelines in the district. There are numerous guidelines for the prevention and management of CVD at both national and international levels. Existent international guidelines are keen on the necessity of smoking cessation, weight optimisation and the importance of physical activity [15]. Among the reasons why CVD are persistent despite the consensus existence of treatment, guideline is the determinant of risk behaviours [15]. The present study identified that behaviour change communication in communities can be a viable opportunity to minimise CVD. Health education is a common expert lead strategy used to address modifiable risk factors for CVD at the community level. In a similar study, practitioners suggested changing direction from case treatment to health promotion by focusing on prevention through awareness creation and community empowerment campaigns to inform the populace about adverse risks factors such as smoking, obesity, and physical inactivity [16]. They also noted that, though such approaches have proved instrumental in CVD prevention, they should be tailored to suit the situation to reduce limitations. The role of community health workers (CHWs) or volunteers becomes crucial on community empowerment through health promotion strategies to deal with modifiable risk behaviours. CHWs are effective in CVD prevention programmes at door-to-door household and community levels because they have a sense of belonging, trust, and duty to protect their communities [17].

Participants mentioned the use of modern technological gadgets in the prevention and management of CVD as a good opportunity that should be encouraged. One component of the CVD-care chain is technology, and it encompasses simple essential tools, and more complex laboratory support [18]. Although lack of equipment such as blood pressure monitoring machines and computers for data storage was a challenge, there exist an opportunity in this regard to effectively prevent and manage CVD cases. Some healthcare workers can use computers, but others still need training in the utilisation of modern technology. The deployment and usage of technology in the Capricorn district remains a big opportunity that could be explored to effectively deal with CVD cases. Despite the challenges that the healthcare system face in the light of CVD prevention and management in the district, it was somewhat functional, per participants’ narratives. For instance, there is a well laid out organogram in place at the district health directorate solely for CVD management and prevention. Thus, we identified that a functional healthcare system is a workable avenue to effectively manage and prevent CVD. This is not limited to the availability of health workforce but also access to essential medicines, sustainable health financing and the role of leadership and governance [19].

### Barriers of CVD prevention and management

The current study unearthed prevailing hurdles in the prevention and management of CVD at the Capricorn district in the Limpopo Province of South Africa as well as opportunities that exist to be explored in the fight against CVD. With reference to poor infrastructural development, we identified that healthcare providers in the district operated in facilities that were particularly small, limiting their ability to have designated space(s) dedicated to CVD cases. This meant that facilities were always overcrowded, impinging on clients’ privacy as individuals with various ailments gathered in same waiting rooms and sought treatment from same consulting room(s). The challenge that limited infrastructure pose to the prevention and management of CVD cases on the African continent is not peculiar to South Africa. For instance, evidence from Eastern Uganda has shown that poor healthcare infrastructure impedes the activities of health teams in combating NCDs such as hypertension by limiting the number of clients that can be received and cared for across various health facilities [17].

Lack of medical supplies and equipment were also identified to be a major barrier to the prevention and management of CVDs in the province. This ranged from the lack of basic diagnostic equipment for check-ups to essential medication for the management of CVDs across the selected health facilities [20]. Logistical challenges in healthcare provision seems to be a systemic problem in South Africa. A recent study conducted in the country identified that shortage of medication was one of the paramount barriers to the prevention and management of non-communicable diseases in the country [21]. Our findings are also buttressed by studies in Argentina and Vietnam, countries with similar socio-economic conditions as South Africa, where lack of good quality information systems was reported as a logistical setback in the management of persons with risk factors [22]. Quality health information systems are essential for the strengthening of health care provision [18]. Health information technology (HIT) targets many of the barriers to successful healthcare delivery for CVD prevention and management. Interventions such as electronic medical records system is a vital strategy in improving patient documentation, data capture, inter-provider communication, and follow-up for NCD management [18]. The huge inadequacy of computers in health facilities thus, makes good health data capture and storage an unattainable aspect of CVD care. According to Kien et al. [22], the lack of comprehensive centralized data-sharing systems in health facilities results in insufficient coordination of information between curative and preventive health systems in Vietnam. In this study, the availability of modern technology has been stated as a facilitator although capacity building in computer usage is still needed. In some facilities, electronic data capturing forces healthcare workers to use part of their already small working space for computers, and to store paper records [19].

Inadequacy of staff was identified to be another strong barrier against CVD prevention and management. The poor staffing of health care facilities across the district had a rippling effect on accessibility to primary health care services, as most facilities were compelled to open late and close early. Poor utilisation is negatively associated with rural residence and socio-economic status [16]. This deficiency meant that patients were reluctant to attend for proper diagnosis or follow-up as the workload was too great for the few health personnel. Poor access to primary health care services was largely reported by respondents as a hindrance to the prevention and management of CVD. The study indicated that health literacy in the form of health education is applicable but the behavioural change component in the form of health promotion is grossly lacking. Focusing on fighting the five major risk factors for CVD: smoking, high blood pressure, bad diet (including high saturated and trans-saturated fats; as well as scanty fruit and vegetables), physical inactivity, and obesity demands health promotion behavioural change strategies [23]. Health promotion is about empowerment of the vulnerable groups and it is highly recommended for successful prevention of CVD at a community level [24].

The limitation of the study is that the findings are based only on interviews conducted with primary health care managers. The findings are based on self-report and may therefore differ from actual practice. This limitation can be alleviated by conducting further studies that involve other multidisciplinary team members that are also playing a role in CVD prevention and management.

### Trustworthiness of the study

#### Validity and reliability

Validity and reliability of the study findings was achieved by first ensuring that the study findings were credible. This was done by employing iterative questioning process during interviewing as well as frequent client probing (Additional file 1: Table 1; [25]).

#### Transferability, confirmability, and dependability

We ensured that our findings could be transferrable and thus could be applied to other contexts with similar characteristics based on the clear and thorough explanation of our methods (26). This also ensured that our study findings are dependable as our methods could easily be used to replicate the study should the need arise. Lastly, we ensured confirmability of our study findings by revisiting our participants with the transcripts and the initial findings for them to confirm that what we have reported was indeed what they narrated during the interview process [26]. This also ensured that the results are the experiences and ideas of the participants, rather than the characteristics and preferences of the team of researchers [27].

## Conclusion

The facilitators and barriers that affect the prevention and management of CVD were identified at the Polokwane Municipality in Limpopo Province. Various stakeholders in the district should, therefore, team up to maximise the facilitators that exist in the district for CVD prevention. For instance, adequate working space should be provided progressively among all healthcare facilities dealing with CVD cases while at the same time equipping them with adequate resources and technology to be able to diagnose and manage CVD cases on time. The study concludes with the strong recommendation that improvement of prevention and management of CVD depends on strengthening facilitators and redressing barriers identified. The prevention of CVD should be focused on primary prevention against adverse risks factors such as smoking, obesity, and physical inactivity at a community level. The role of community health workers should be strengthened on community empowerment through health promotion strategies to deal with modifiable risk behaviours among the people in their households and communities.

## Supplementary Information

**Additional file 1**. Qualitative interview guide domains and examples.

## Data Availability

Data is available upon request by contacting the corresponding author. The data that support the findings of this study are available from European Commission (SPICES project) but restrictions apply to the availability of these data, which were used under license for the current study, and so are not publicly available. Data are however available from the authors upon reasonable request and with permission of European Commission (SPICES project).

## Abbreviations

AM: Area manager
CHW: Community health worker
CVD: Cardiovascular disease
KI: Key informant
OPM: Operational manager
NCDs: Non communicable diseases
NGO: Nongovernmental organisations
PHC: Primary health care
PI: Principal investigator
WHO: World Health Organisation

## References

[1] Mbewu A (2015). The burden of cardiovascular disease in Sub-Saharan Africa. Eur Heart J.

[2] Nojilana B, Pillay-Van Wyk V, William M, Somdyala N, Joubert JD, Groenewald P (2016). Persistent burden from non-communicable diseases in South Africa needs strong action. South Afr Med J.

[3] Bigna JJ, Noubiap JJ (2019). The rising burden of non-communicable diseases in sub-Saharan Africa. Lancet Global Health.

[4] WHO. Cardiovascular diseases (CVDs) Factsheet. May 2017 [Internet]. 2017. Available from: https://www.who.int/en/news-room/fact-sheets/detail/cardiovascular-diseases-(cvds).

[5] Roth GA, Forouzanfar MH, Moran AE, Barber R, Nguyen G, Feigin VL (2015). Demographic and epidemiologic drivers of global cardiovascular mortality. N Engl J Med.

[6] Ndinda C, Ndhlovu TP, Juma P, Asiki G, Kyobutungi C (2018). The evolution of non-communicable diseases policies in post-apartheid South Africa. BMC Public Health.

[7] Zühlke L (2018). The future of cardiovascular disease in South Africa and the role of the South African Heart Association. SA Heart.

[8] Cappuccio FP, Miller MA (2016). Cardiovascular disease and hypertension in sub-Saharan Africa: burden, risk and interventions. Intern Emerg Med.

[9] Wändell PE, de Waard AM, Holzmann MJ (2018). Barriers and facilitators among health professionals in primary care to prevention of cardiometabolic diseases: a systematic review. Fam Pract.

[10] Mayosi BM, Flisher AJ, Lalloo UG, Sitas F, Tollman SM, Bradshaw D (2009). The burden of non-communicable diseases in South Africa. Lancet.

[11] National Government of South Africa (ZA). Municipalities in South Africa; Limpopo Municipalities [internet]; South Africa; 2018. Available from: https://www.localgovernment.co.za/provinces/view/5/limpopo.

[12] Beyers JL (2015). Service delivery challenges within municipalities in the capricorn district of Limpopo Province. J Hum Ecol.

[13] Id AA, Dodoo F, Awuah RB, Owusu-dabo E, Addo J, Nicolaou M, et al. Knowledge and perceptions of type 2 diabetes among Ghanaian migrants in three European countries and Ghanaians in rural and urban Ghana: the RODAM qualitative study. PLoS ONE. 2019;1–23. 10.1371/journal.pone.0214501 PMC6445464 30939148

[14] Saunders B, Sim J, Kingstone T, Baker S, Waterfield J, Bartlam B (2018). Saturation in qualitative research: exploring its conceptualization and operationalization. Qual Quant.

[15] Stewart J, Manmathan G, Wilkinson P. Primary prevention of cardiovascular disease: a review of contemporary guidance and literature. JRSM Cardiovasc Dis.2017;1–9. 10.1177/2048004016687211 PMC5331469 28286646

[16] Chikafu H, Chimbari MJ (2019). Cardiovascular disease healthcare utilization in Sub-Saharan Africa: a scoping review. Int J Environ Res Public Health.

[17] Ojo TT, Hawley NL, Desai MM, Akiteng AR, Guwatudde D, Schwartz JI (2017). Exploring knowledge and attitudes toward non-communicable diseases among village health teams in Eastern Uganda: a cross-sectional study. BMC Public Health.

[18] Jabbour S, Nishtar S, Prabhakaran D, Chockalingam A, Achutti A, Agrawal A (2003). Information and communication technology in cardiovascular disease prevention in developing countries: hype and hope. Report of the international collaboration on information use in cardiovascular health promotion in developing countries. Int J Cardiol.

[19] Mercer T, Njuguna B, Bloomfield GS, Dick J, Finkelstein E, Kamano J (2019). Strengthening referral networks for management of hypertension across the health system (STRENGTHS) in western Kenya: a study protocol of a cluster randomized trial. Trials.

[20] Cheong AT, Khoo EM, Liew SM, Chinna K (2018). What are the determinants for individuals to undergo cardiovascular disease health checks? A cross sectional survey. PLoS ONE.

[21] Ntuli ST, Maimela E, Alberts M, Choma S, Dikotope S, Africa S, et al. Prevalence and associated risk factors of hypertension amongst adults in a rural community of Limpopo Province, South Africa. Afr J Primary Health Care. 2015;1–5. 10.4102/phcfm.v7i1.847 PMC4685651 26842512

[22] Kien VD, Van Minh H, Giang KB, Ng N, Nguyen V, Tuan LT (2018). Views by health professionals on the responsiveness of commune health stations regarding non-communicable diseases in urban Hanoi, Vietnam: a qualitative study. BMC Health Serv Res.

[23] Egger G, Spark R, Lawson J, Donovan R (2013). Health promotion strategies & methods.

[24] WHO. Ottawa Charter for Health Promotion, First International Conference on Health Promotion. 1986;1–4. Available from: http://www.who.int/healthpromotion/conferences/previous/ottawa/en/.

[25] Noble H, Smith J. Issues of validity and reliability in qualitative research. Evid Based Nurs. 2015;34–5. 10.1136/eb-2015-102054 25653237

[26] Lincoln Y, Guba E (2005). Naturalistic inquiry.

[27] Shenton AK (2004). Strategies for ensuring trustworthiness in qualitative research projects. Educ Inf.

