# Supplementary material for: Facilitators and barriers in prevention of cardiovascular disease in Limpopo, South Africa: a qualitative study conducted with primary health care managers
Source: BMC Cardiovasc Disord. 2021 Oct 12;21:492. doi: 10.1186/s12872-021-02290-1 (PMC8507214; doi:10.1186/s12872-021-02290-1)
Supplement: Supplementary file 1 — Additional file 1. Qualitative interview guide domains and examples. [file 12872_2021_2290_MOESM1_ESM.docx]

**Qualitative interview guide domains and examples**

| **Domains** | **Primary and secondary items** |
| --- | --- |
| Burden of cardiovascular diseases in country/ study site | - What are the common chronic diseases reported in this area? Probe: Ranking of CVDs among chronic diseases; the main CVDs documented - What are the main vulnerable groups for CVDs at this district/region/population and what are their healthcare needs/concerns? Who is not reached? |
| Administrative and Policy Environment | - Is there any national/regional/district policy for CVD prevention, care and management? (List the documents) - Is there a national or district health plan that addresses CVD prevention? Probe: What is included in the plan? Who provides CVD prevention, care and management? - How is the health care system organized in terms of CVD prevention? - What are the main problems in the current health care system? (Health personnel? health programme planning, programme implementation/management, health facilities/infrastructure, health care supplies)? |
| Opportunities and barriers in CVD prevention and management in South Africa | - Health Promotion in relation to CVDs: Is there any health promotion activities for CVDs? Probe: Who does it, where, when and how is it done within and outside the facilities—community outreaches; who is targeted by the promotion; what are its successes and challenges? - CVD profiling: Is profiling done (at facility, community level, etc.). Probe: how is it done (characterization based on risk factors, diagnosis, etc.), by who? What tools are used? - Care and Management/treatment: Current policies, guidelines, and procedures—who reviews the clients/patients, makes diagnosis, investigates, and prescribes medications? - Follow-up and self-management: What is the schedule of follow-ups, how are patients that do not return for appointments detected and tracked, how is the adherence to interventions/care monitored, by who, what is the current retention of patients on treatment, what are the challenges and successes; What self-management strategies are currently being implemented? - How do you think your healthcare/organization's culture (general beliefs, values, assumptions that people embrace, code of conduct, policies) affect the implementation of CVD programs? - To what extent do current CVD programs at the health facility/primary healthcare fail to meet existing population needs? - How do stakeholders (consumers, providers, and policymakers) feel about current programs/practices/process that are available related to CVD prevention, care, and management at this facility? |
